# The callus formation capacity of strawberry leaf explants is modulated by DNA methylation

**DOI:** 10.1093/hr/uhab073

**Published:** 2022-01-19

**Authors:** Decai Liu, Qin Mu, Xianyang Li, Sheng Xu, Yi Li, Tingting Gu

**Affiliations:** 1 State Key Laboratory of Plant Genetics and Germplasm Enhancement and College of Horticulture, Nanjing Agricultural University, Nanjing, 210095 China; 2 Department of Plant Science and Landscape Architecture, University of Connecticut, Storrs, CT 06269, USA

## Abstract

Shoot regeneration from leaf tissue requires the de-differentiation of cells from a highly differentiated state into an actively dividing state, but it remains unclear how this physiological transition occurs and is regulated, especially at the epigenetic level. Here, we characterized the DNA methylome represented by 5-methylcytosine (5mC) in leaf and callus tissue derived from leaf explants of woodland strawberry, *Fragaria vesca*. We detected an overall increase in DNA methylation and distinct 5mC enrichment patterns in the CG, CHG, and CHH sequence contexts in genes and transposable elements. Our analyses revealed an intricate relationship between DNA methylation and gene expression level in leaves or leaf-derived callus. However, when considering the genes involved in callus formation and shoot regeneration, *e.g. FvePLT3/7*, *FveWIND3*, *FveWIND4*, *FveLOG4* and *FveIAA14*, their dynamic transcription levels were associated with differentially methylated regions located in the promoters or gene bodies, indicating a regulatory role of DNA methylation in the transcriptional regulation of pluripotency acquisition in strawberry. Furthermore, application of the DNA methyltransferase inhibitor 5′-azacytidine (5′-Aza) hampered both callus formation and shoot regeneration from the leaf explants. We further showed that 5′-Aza downregulated the expression of genes involved in cell wall integrity, such as *expansin*, *pectin lyase*, and *pectin methylesterase* genes, suggesting an essential role of cell wall metabolism during callus formation. This study reveals the contribution of DNA methylation to callus formation capacity and will provide a basis for developing a strategy to improve shoot regeneration for basic and applied research applications.

## Introduction

Plants are able to reprogram highly differentiated cells to regenerate new individuals, as apical meristems are reconstructed when plants are partially damaged. In vitro organogenesis can be initiated by culturing isolated organs or tissues in a medium supplemented with auxin and cytokinin. A higher auxin:cytokinin ratio triggers callus formation, whereas a higher cytokinin:auxin ratio mediates the re-designation of cell fate and establishes meristems [1]. The *de novo* regeneration process not only provides an ideal model for studying the mechanisms underlying plant cell differentiation and organogenesis but also is pivotal for the application of plant biotechnology to crop breeding programs in the horticulture community [2]. Some model plants are able to produce stable transgenic plants through induction of *in vitro* regeneration via tissue culture [3, 4]. However, efficient regeneration systems for propagation and genetic transformation have not been established for many horticultural crops.

Studies in Arabidopsis demonstrate that changes in cell fate during callus formation are regulated by transcription factors (TFs) under hormonal and environmental control. During callus induction in auxin-rich media, the LBD (LATERAL ORGAN BOUNDARIES DOMAIN) family genes play an essential role in mediating auxin signals to activate *E2 PROMOTER BINDING FACTOR a* (*E2Fa*) [5, 6]. bZIP59 forms a complex with LBD proteins to activate *FAD-BD* to regulate auxin-induced callus formation [7]. In addition, transcription factors encoded by *type-B ARR* (*ARR-B*) genes, *APETALA2/ETHYLENE RESPONSE FACTOR* (*AP2/ERF*) genes and *ENHANCER OF SHOOT REGENERATION 1* (*ESR1*) and *ESR2* genes activate the expression of D-type cyclin genes in cell cycle control to promote cytokinin-mediated callus formation [8]. The callus and shoot formation processes induced by wounding require the activation of *WOUND-INDUCED DEDIFFERENTIATION 1* (*WIND1*), *WIND2*, *WIND3*, and *WIND4*, genes belonging to another AP2/ERF family. WIND proteins upregulate the cytokinin response mediated by ARR-B and ESR1 proteins to promote dedifferentiation and cell division [9–11]. Furthermore, some genes associated with embryogenesis or meristem development, including *WUSCHEL* (*WUS*), *WUSCHEL-RELATED HOMEOBOX 11* (*WOX11*), *SHOOT MERISTEMLESS* (*STM*) and *ELONGATED HYPOCOTYL5* (*HY5*), have also been reported to control callus formation [12].

The dedifferentiation process of plant cells involves the dynamic decondensation of chromatin [13, 14]. DNA methylation is actively involved in the regulation of gene transcription and chromatin remodeling in eukaryotic cells. In plants, 5-methylcytosine (5mC) is a widespread DNA modification that occurs in CG, CHG, and CHH (where H = A, T, or C) sequence contexts [15]. Changes in DNA methylation in cell culture were reported about thirty years ago in Napier grass [16]. Recently, dramatic changes in DNA methylation have been reported in these three cytosine sequence contexts along genes and transposable elements (TEs) during callus formation in Arabidopsis [17], sugar beet [18], and other plant species [19]. In Arabidopsis, the double mutant of *de novo* methyltransferases *drm1/drm2* produces larger and heavier callus from both explants and seedlings compared with the wild-type plant [20]. *WUS* is silenced by DNA METHYLTRANSFERASE 1 (MET1), and its activation for shoot regeneration is modulated by a reduction in DNA methylation in the promoter sequences [21, 22]. Accumulating evidence indicates that the global and local chromatin status modulated by DNA methylation is crucial for the regulation of cell differentiation and dedifferentiation [13, 19, 21, 23].

Elucidation of the molecular mechanisms that underlie callus formation in tissue culture is crucial for the establishment of an efficient *in vitro* regeneration system. To study the regulatory role of DNA methylation in callus formation, we profiled the DNA methylomes of leaf tissue and leaf-derived callus by whole genome bisulfite sequencing (WGBS). We found an overall increase in DNA methylation and distinct 5mC enrichment patterns in the three cytosine sequence contexts in genes and TEs. Furthermore, we revealed a close association between the enrichment level of DNA methylation and the transcription level of genes involved in callus formation and shoot regeneration. Finally, we found that application of the DNA methyltransferase inhibitor 5′-azacydine (5′-Aza) reduced both callus formation and shoot regeneration from leaf explants, and we proposed that the inhibitory effect of 5′-Aza resulted from decreased transcript levels of genes related to cell wall integrity. This study characterizes the DNA methylome during callus formation in *F. vesca* and provides clues for improving the regeneration efficiency of strawberry and other Rosaceae species.

## Results

### Dynamic DNA methylation landscape during callus formation from leaf explants in *F. vesca*

**Figure 1:**
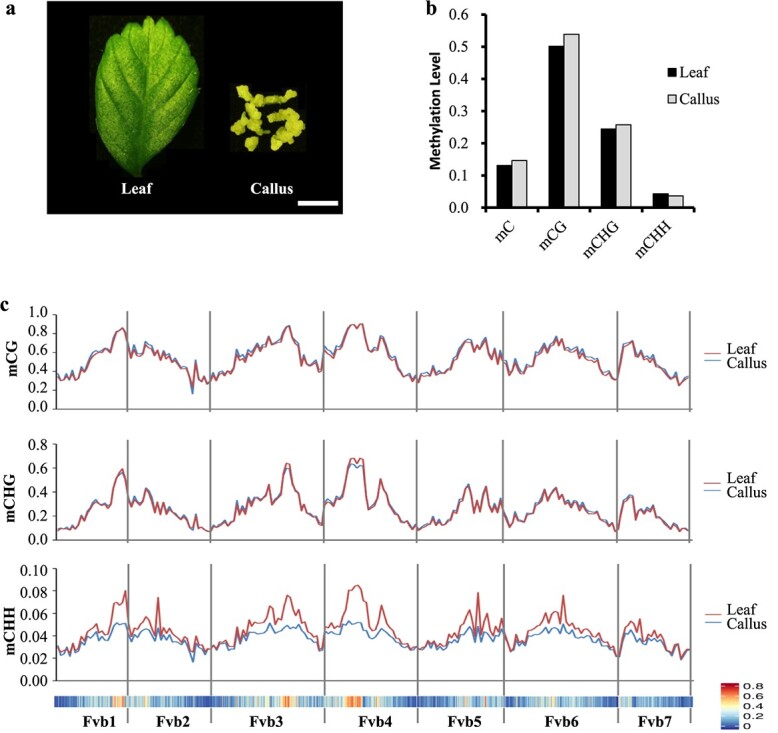
The genome-wide DNA methylation pattern during callus formation from leaf explants in woodland strawberry. (a) Illustration of the leaf and leaf-derived callus collected for sequencing. Leaves (30–35 d old) from 50–60-d-old seedlings were used as explants, and leaf-derived callus was collected from leaf explants cultured on callus induction medium for 30 d. Scale bar = 0.5 cm. (b) The average methylation levels of mC, mCG, mCHG and mCHH in leaf and leaf-derived callus tissues. (c) Chromosomal distribution of mCG, mCHG and mCHH in leaf and leaf-derived callus tissues. The red and blue lines represent leaf and leaf-derived callus, respectively. The bottom portion is a heat map of TE enrichment along each chromosome.

To reveal the DNA methylation landscape during the dedifferentiation process in woodland strawberry, *F. vesca*, bisulfite sequencing was performed on leaf tissue and leaf-derived callus (Fig. 1a). Two independent biological replicates of the DNA methylome were generated, yielding an average sequencing depth of 30× for each sample (Table S1).

In leaf tissues, 13.13% of the total cytosine sites were methylated (Fig. 1b). The average 5mC levels in the CG, CHG, and CHH sequence contexts were 50.14%, 24.44%, and 4.30%, respectively. In callus, 14.63% of the total cytosine sites were methylated, a higher percentage than in leaf tissue, and the average 5mC levels in the three cytosine sequence contexts were 53.86%, 25.77%, and 3.66%, respectively (Fig. 1b). Notably, during the dedifferentiation process, the overall DNA methylation enrichment in the symmetric contexts of CG and CHG increased, whereas DNA methylation enrichment decreased in the asymmetric CHH context in leaf-derived callus compared to leaves (Fig. 1b, c), indicating diversified regulatory mechanisms in different sequence contexts. Chromosome-wide profiles further demonstrated that the enrichment levels of mCG, mCHG, and mCHH were all positively correlated with local TE density in both leaf and callus (Fig. 1c). Although the overall mCHG level increased in callus, the mCHG level in TE-rich sequences decreased. Moreover, the enrichment level of mCHH also decreased significantly in TE-rich regions.

### DNA methylation enrichment patterns in genes and TEs

**Figure 2:**
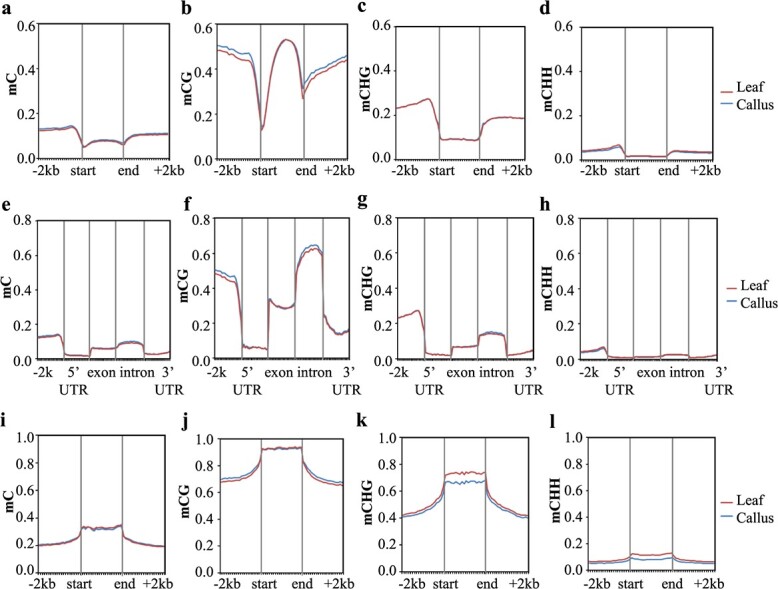
Characterization of the DNA methylation pattern along genes and TEs in leaf and leaf-derived callus tissues. (a–d) Distribution of mC, mCG, mCHG and mCHH along genes. The enrichment patterns within the gene body and 2 kb upstream (−2 kb) and downstream (+2 kb) are profiled. (e–h) Distribution of mC, mCG, mCHG and mCHH in various genic elements, including the promoter (2 kb upstream of the TSS), 5′-UTR, exon, intron and 3′-UTR. (i–l) Distribution of mC, mCG, mCHG and mCHH along the TE body and 2 kb upstream (−2 kb) and downstream (+2 kb). The red and blue lines represent leaf and leaf-derived callus, respectively.

Significant variations in DNA methylation changes along genic regions or other functional elements during dedifferentiation have been observed in several plant species. We plotted the average methylation levels along gene bodies by metagene analysis (Fig. 2a–d) and revealed different distribution patterns of mCG, mCHG and mCHH. In both leaves and leaf-derived callus, mCG levels in genic regions were comparable to those in the surrounding intergenic regions, with a sharp dip around the transcriptional start site (TSS) and the transcriptional end site (TES). By contrast, mCHG and mCHH levels were relatively depleted in genic regions compared to the surrounding sequences (Fig. 2a–d). A detailed analysis of the various functional genic elements revealed that DNA methylation was most enriched in introns and promoters, lower in exons, and most depleted in the 5′UTR and 3′UTR in all CG, CHG, and CHH contexts (Fig. 2e–h).

Notably, DNA methylation enrichment in different sequence contexts showed various dynamic patterns during callus formation (Fig. 2e–h). The overall mCG level did not change in exons, 5′UTRs, or 3′UTRs but increased slightly in intronic and intergenic sequences (Fig. 2b, f). The overall mCHG enrichment did not change significantly in either genic or intergenic sequences, except that mCHG in introns increased in callus relative to leaf tissue (Fig. 2c, g). Lastly, the enrichment level of mCHH decreased in intergenic regions, in contrast to the changes in mCG and mCHG (Fig. 2d, h).

We next characterized the methylation patterns in various classes of TEs. Overall, DNA methylation in the three cytosine sequence contexts had similar patterns in leaf and callus: mCG, mCHG, and mCHH were all more enriched in TE bodies than in the surrounding sequences (Fig. 2i–l). Meanwhile, various TE classes had different enrichment levels of DNA methylation. Among the TEs investigated, Class I long terminal repeats (LTRs, exemplified by Copia and Gypsy) and Class II DNA transposons had the highest enrichment levels of mCG and mCHG in their TE bodies, whereas SINEs belonging to Class I long terminal repeats had the highest level of mCHH (Fig. S1). In addition, in the sequences surrounding TEs, mCHG and mCHH levels decreased significantly around Copia and Gypsy but remained relatively stable around LINEs, SINEs, and DNA transposons (Fig. S1).

During callus formation, the dynamic changes in DNA methylation along TEs were different in the three cytosine sequence contexts (Fig. 2j–l). The enrichment of mCG in callus decreased slightly in TE bodies but increased in the upstream or downstream regions (Fig. 2j). By contrast, the enrichment levels of mCHG and mCHH in callus decreased in both TE bodies and the surrounding sequences (Fig. 2k, l). In sum, in contrast to genic sequences, TEs showed an overall decrease in DNA methylation enrichment in callus compared to leaf tissue, and various TE classes had TE type-specific and sequence context-specific distribution patterns.

### Transcript levels of DNA methyltransferase and demethylase genes are associated with DNA methylation patterns

**Figure 3:**
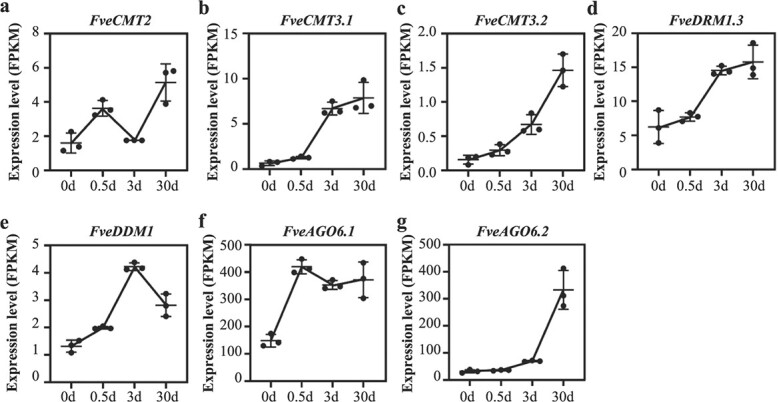
Expression patterns of genes mediating DNA methylation during callus formation. The profiled genes include the DNA methyltransferase genes *FveCMT2*, *FveCMT3.1*, *FveCMT3.2* and *FveDRM1.3* (a–d), the chromatin-remodeling factor gene *FveDDM1* (e) and the siRNA-guided DNA methylation pathway genes *FveAGO6.1* (f) and *FveAGO6.2* (g). On the x-axis, 0 d, 0.5 d, 3 d and 30 d refer to leaf tissue, leaf explants cultured on callus induction medium for 12 h or 3 d, and callus collected from leaf explants cultured on callus induction medium for 30 d, respectively. Error bars denote means ± SD, n = 3.

The local and overall enrichment patterns of DNA methylation are balanced by the methylation and demethylation processes [24–26]. Transcript levels of genes whose products mediate DNA methylation and demethylation were analyzed to investigate the possible mechanisms underlying differences in the DNA methylome during callus formation (Fig. 3 and Fig. S2). Four out of the eight DNA methyltransferase genes were upregulated 2.2- to 12.0-fold in callus relative to leaf tissue. These included *FveCMT2*, *FveCMT3.1*, and *FveCMT3.2*, which participate in mCHG maintenance, and *FveDRM1.3*, which is associated with *de novo* cytosine methylation in all sequence contexts (Fig. 3a–d and Table S2). In addition, *FveDDM1*, a chromatin remodeling gene indispensable for maintaining genome-wide DNA methylation, was also marginally upregulated (Fig. 3e). Of the genes involved in RNA-dependent DNA methylation (RdDM), the transcripts of two *FveAGO* genes (*FveAGO6.1* and *FveAGO6.2*, which are *AtAGO6* orthologs) increased 2.5- to 10.5-fold in callus relative to leaves (Fig. 3f, g and Table S2). On the other hand, none of the four DNA demethylase genes were differentially expressed between leaf and callus tissue (Table S2). In sum, four DNA methyltransferase genes and two *AGO* genes were upregulated in leaf-derived callus relative to leaf tissue, consistent with the overall increase in DNA methylation enrichment observed during the formation of callus from leaf explants.

### Co-analysis of DNA methylation enrichment and gene expression level

**Figure 4:**
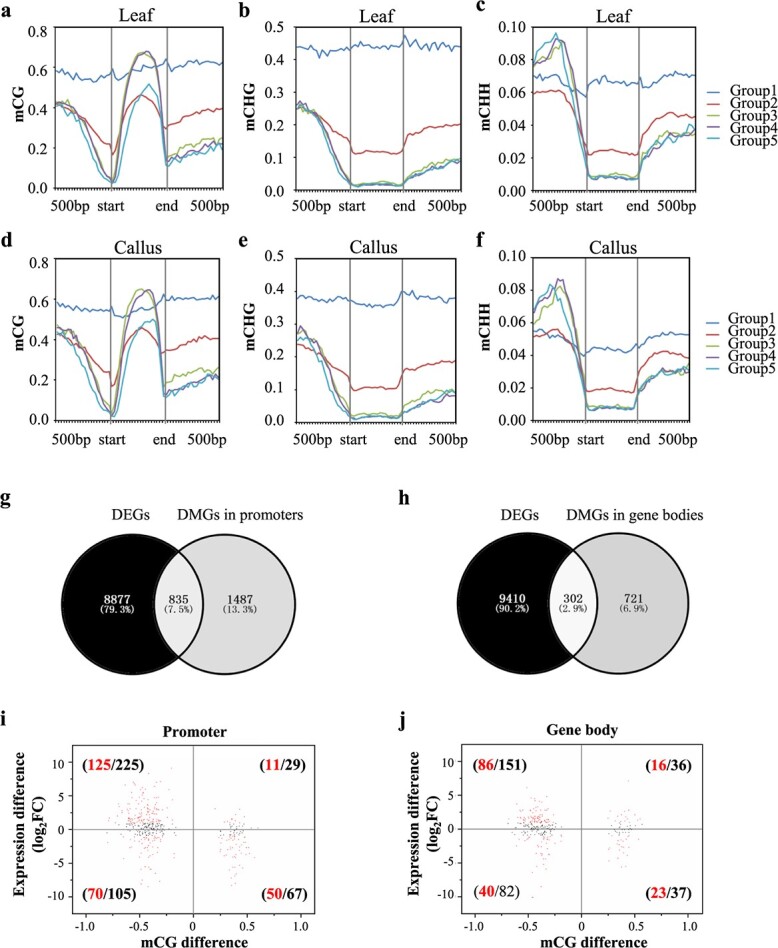
Genome-wide co-analysis of DNA methylation enrichment and gene expression level. (a–f) Genes are classified into five groups according to their transcription levels (Group1: FPKM = 0, Group2: 0 < FPKM ≤ 5, Group3: 5 < FPKM ≤ 10, Group4: 10 < FPKM ≤ 30, Group5: FPKM > 30), and their DNA methylation levels are profiled. (g–h) Venn diagrams illustrating the overlap between differentially expressed genes (DEGs) and DMR-associated genes (DMGs) during callus formation. DMGs with DMRs in their promoters (2 kb upstream of the TSS) or gene bodies are profiled separately. The overlapping DEGs and DMGs are defined as methylDEGs. (i–j) Association analysis between changes in gene expression level and changes in mCG in promoters (i) and gene bodies (j) during callus formation. The x-axis is the change in mCG (only genes with |ΔmCG| ≥ 0.15 are plotted, black points). The y-axis represents the gene expression difference (log_2_FC). The red points are the DEGs that satisfy |ΔmCG| ≥ 0.15, |log_2_FC| ≥ 1 and *P_adj_* < 0.05, and the numbers in black and red refer to the number of black and red points in each quadrant.

Although DNA methylation has been revealed as a factor that modulates the transcription of some plant genes, it remains unclear whether it has a general regulatory effect. To study the correlation between DNA methylation and gene transcript level in strawberry, we first classified the genes into five groups as silent (FPKM = 0, group 1), expressed at a low level (0 < FPKM ≤ 5, group 2), expressed at medium levels (5 < FPKM ≤ 10, group 3 and 10 < FPKM ≤ 30, group 4) and expressed at a higher level (FPKM > 30, group 5), and we then profiled the enrichment of DNA methylation accordingly. The distribution pattern of DNA methylation in the CG, CHG, and CHH contexts was similar in leaves and leaf-derived callus (Fig. 4a–f). In the profiled gene-flanking regions, 500 bp upstream of the TSS or downstream of the TES, mCG and mCHG levels were higher in genes with lower expression than in genes with medium and higher expression but lower than in silent genes (Fig. 4a, b, d, e). mCHH levels were higher in genes with medium or higher expression than in genes with lower or silent expression in promoter sequences but decreased significantly around the TSS (Fig. 4c and 4f). Similarly, the DNA methylation distribution pattern in the three cytosine sequence contexts varied in the gene body. In the CG context, genes with medium expression had higher mCG levels than those with lower or higher expression (Fig. 4a, d). In the CHG and CHH contexts, genes with higher or medium expression had lower mCHG and mCHH levels than genes with lower expression. Overall, gene expression level was related to DNA methylation in different sequence contexts, but the differences among the genes with medium expression (5 < FPKM ≤ 30) were not distinct.

To further investigate the correlation between DNA methylation enrichment and transcript level during callus formation, differentially expressed genes (DEGs) and differentially methylated region-associated genes (DMGs) were co-analyzed. In total, 9712 DEGs (|log_2_FC| ≥ 1, *P_adj_* < 0.05, FPKM ≥ 1) were identified between leaves and leaf-derived callus, including 5090 upregulated genes and 4622 downregulated genes. DMGs were defined as genes with a differentially methylated region (DMR) within the 2 kb of sequence upstream of the TSS or within the gene body. We identified 2322 and 1023 genes with associated DMRs in the promoter and the gene body, respectively (Fig. 4g and 4h). Among these DMGs, 835 and 302 were also found to be DEGs (Fig. 4g and 4h, defined as methylDEGs). An association study between DNA methylation and transcript level revealed that among the 330 DMGs with mCG loss in promoters in callus relative to leaf, the transcript levels of 37.9% (125/330) and 21.2% (70/330) were up- and downregulated, respectively (Fig. 4i). In addition, among the 233 DMGs with mCG loss in gene bodies, the transcription levels of 36.9% (86/233) and 17.2% (40/233) were up- and downregulated, respectively (Fig. 4i). These results indicated a very weak negative correlation between transcription and mCG level in both the promoter and the gene body. However, this weak correlation was not observed in the CHG or CHH sequence contexts (Fig. S2). Thus, the association between DNA methylation enrichment and gene expression level is intricate during the formation of callus from leaf explants in strawberry.

### Negative correlation between DNA methylation enrichment and transcript levels of callus formation-related genes

Gene Ontology (GO) enrichment analyses of the methylDEGs revealed that the up- and downregulated methylDEGs were most enriched in the terms catalytic activity and oxidoreductase activity (Fig. S3). Kyoto Encyclopedia of Genes and Genomes (KEGG) analyses found that the up- and downregulated methylDEGs were enriched in pathways involved in metabolism, *e.g.* biosynthesis of secondary metabolites, cysteine, and methionine metabolism, and fatty acid degradation (Fig. S4). These analyses suggest that DNA methylation may be involved in complicated physiological and biochemical processes during callus formation.

**Figure 5:**
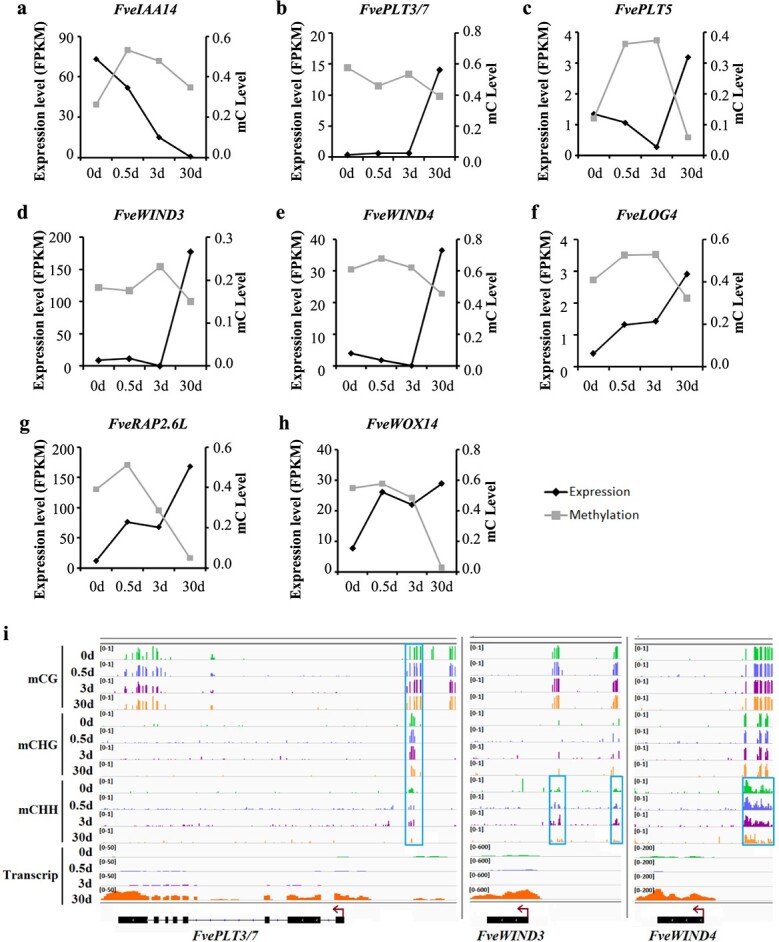
Correlation between DNA methylation enrichment and gene expression level of genes related to callus formation and shoot regeneration. (a–h) Negative correlations between DNA methylation and transcript level of genes essential for callus formation and shoot regeneration, *e.g. FveIAA14* in the hormonal pathway and *FveWIND3* in the wounding pathway. On the x-axis, 0 d, 0.5 d, 3 d and 30 d refer to leaves, leaf explants cultured on callus induction medium for 12 h or 3 d, and leaf-derived callus collected from leaf explants cultured on callus induction medium for 30 d, respectively. The y-axes on the left and right indicate gene expression level (FPKM) and DNA methylation level (mC), respectively. (i) IGV browser shots showing the DNA methylation enrichment along the genic and promoter sequences of *FvePLT3/7*, *FveWIND4* and *FveWIND4*. The blue boxes indicate differentially methylated regions between leaf and leaf-derived callus tissue, and the brown arrows represent the direction of gene transcription.

Some hormone-related and wounding-responsive pathway genes are essential for *de novo* organogenesis in Arabidopsis. We identified the *F. vesca* genes orthologous to key genes involved in callus formation and analyzed their transcript levels (Table S3). Among the IAA-ARF-LBD-E2Fa pathway genes that trigger the formation of callus in auxin-rich callus induction medium, the negative regulator *FveIAA14* was downregulated 112.7-fold (Fig. 5a and Table S3), whereas some positive regulators, *e.g. FveLBD16*, *FveE2Fa* and *FvePME*, were upregulated 2.1- to 22.7-fold in callus relative to leaves (Table S3). The transcript levels of *FvePLT3/7* and *FvePLT5*, which activate *FveCUC1* and *FveCUC2* for the acquisition of pluripotency in growing callus, also increased 2.4- to 39.1-fold (Fig. 5b, c and Table S3). Some TFs downstream of wounding signals for callus induction, *FveWIND3* and *FveWIND4*, were upregulated 9.1- and 19.9-fold, respectively (Fig. 5d, e and Table S3). The cytokinin biosynthesis genes *FveIPT3* and *FveLOG4*, which are activated by wounding, were up-regulated 3.6- and 7.1-fold, respectively (Table S3 and Fig. 5f). Three cyclin genes essential for cell proliferation, *FveCYCD3.1*, *FveCYCD3.2*, and *FveCYCD3.3*, were upregulated 1.9- to 17.6-fold (Table S3). Furthermore, the transcript levels of some regeneration-related genes, *e.g. FveRAP2.6 L*, *FveWOX14*, *FveWUS*, *FvePHV* and *FveESR1*, were also enhanced in callus tissues (Fig. 5g, h, Table S3). Overall, dozens of genes that play a regulatory role in the dedifferentiation and regeneration processes of Arabidopsis showed a similar expression pattern during callus formation in *F. vesca*, suggesting convergence of the gene regulatory network in the two plant species.

**Figure 6:**
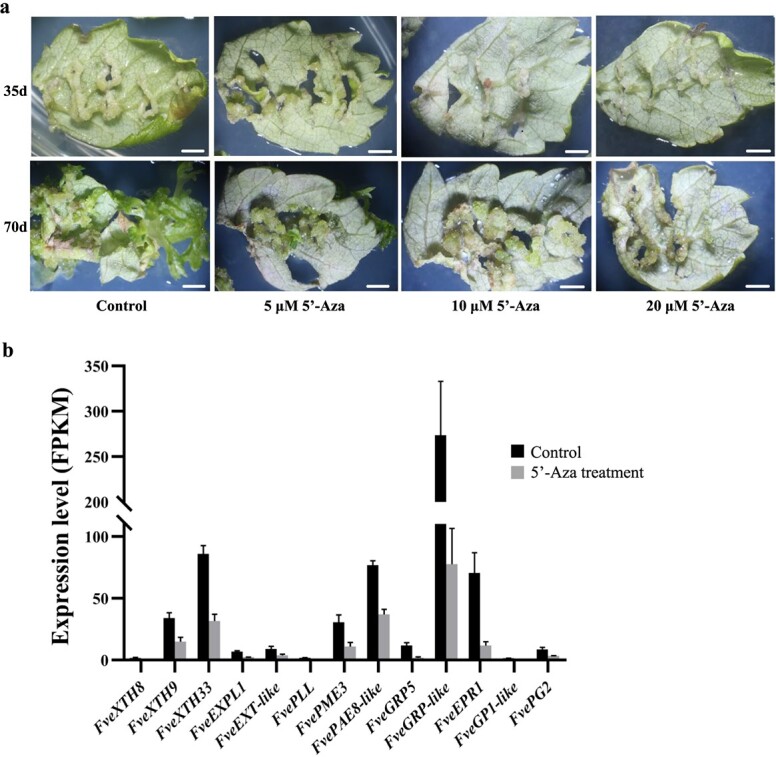
The negative effect of the DNA methyltransferase inhibitor 5′-Aza on callus formation. (a) Application of 5′-Aza to the callus induction medium inhibits callus formation and shoot regeneration. The upper four pictures show the state of callus formation for different concentrations of 5′-Aza on day 35, and the lower four pictures show the state of shoot regeneration for different concentrations of 5′-Aza on day 70. As the concentration of 5′-Aza increases, the capacity for callus formation and shoot regeneration decreases. Scale bar = 0.2 cm. (b) Transcription levels of some genes related to cell wall integrity decrease upon 5′-Aza treatment. Leaf tissues incubated on callus induction medium supplemented with 0 or 20 μM 5′-Aza for 3 d were collected for sequencing.

Next, dynamic changes in DNA methylation were profiled for the genes involved in dedifferentiation and regeneration. Out of 31 DEGs putatively related to callus formation and shoot regeneration, 11 were associated with changes in DNA methylation (Table S3). In total, 8 of these 11 methylDEGs showed a negative correlation between gene expression changes and DNA methylation changes during callus formation (Fig. 5a–h), indicating a role of the DNA methylation machinery in their expression. For example, in the promoter region of *FvePLT3/7*, two DMRs in the CG and CHH contexts were detected, together with a decrease in the overall mC level (Fig. 5i). The increased transcript levels of *FveWIND3* and *FveWIND4* were associated with the detection of a DMR in the CHH context upstream of their TSSs (Fig. 5i). In addition, the changed expression levels of *FvePLT5*, *FveRAP2.6 L*, *FveIAA14*, *FveLOG4*, and *FveWOX14* were all negatively correlated with the overall mC enrichment levels in their promoters or gene bodies (Fig. S5), indicating a role of DNA methylation in their transcriptional regulation during callus formation.

### The effect of 5′-Aza on callus formation

Leaf explants were treated with the DNA methyltransferase inhibitor 5′-Aza at concentrations of 0 (control), 5, 10, and 20 μM to study the effect of DNA methylation on callus formation (Fig. 6a). During incubation on callus induction medium, callus tissues grew out along the cut edges of control leaves, but the application of 5′-Aza inhibited callus formation and subsequent shoot regeneration (Fig. 6a), suggesting a role for DNA methyltransferase in this process.

We profiled the transcriptomes of leaf tissues treated with 20 μM 5′-Aza for 12 h or 3 d to investigate the mechanisms underlying the inhibition of callus formation capacity by 5′-Aza. A total of 361 DEGs were identified 12 h or 3 d after 5′-Aza application, including 157 upregulated genes and 204 downregulated genes (Table S4). Among the 204 genes downregulated by 5′-Aza, 78 were also upregulated in callus relative to leaves (Table S5), suggesting their possible involvement in callus formation. Functional annotation of these 78 genes identified 13 genes involved in cell wall metabolism, including *expansin*, *xyloglucan endotransglucosylase*, *pectin lyase*, and *pectin methylesterase* genes (Fig. 6b, reduced 2.1- to 6.3-fold). Some genes essential for cell wall degradation have been reported to regulate callus initiation [27]. Thus, the inhibitory effect of 5′-Aza on callus formation may result from reductions in the transcript levels of genes associated with cell wall degradation. The 78 genes downregulated by 5′-Aza also included several hormone signaling pathway genes that are responsive to gibberellin, auxin, and ethylene, including four gibberellin-responsive genes (FvH4_2g04680, FvH4_2g33410, FvH4_2g38480, and FvH4_6g50260), the auxin-responsive gene *FveIAA33*, and the ethylene-responsive TF *FveTINY2*, which were downregulated 3.1- to 114.7-fold (Table S5). Expression of some other transcription factor genes was reduced 2.1- to 5.0-fold upon 5′-Aza treatment as well: *WUSCHEL-related homeobox 4*, two *bHLHs*, one zinc finger family gene, and one *WRKY* gene (Table S5). In sum, dozens of genes in the pathways of cell wall integrity and hormone signaling were downregulated by the inhibition of DNA methyltransferases in leaf explants, which may have resulted in the inhibitory effect of 5′-Aza on callus formation and shoot regeneration.

## Discussion

DNA methylation plays a significant role in regulating the genes associated with *in vitro* morphogenetic competence in Arabidopsis. The impact of DNA methylation on callus formation has not been fully explored in horticultural crops. In this work, we systematically characterized the 5mC DNA methylome by WGBS during the formation of callus from leaf explants of woodland strawberry, *F. vesca*, and explored the possible regulatory role of DNA methylation in callus formation. We found an overall increase in DNA methylation and distinct 5mC enrichment patterns in the CG, CHG, and CHH sequence contexts in genic regions and transposable elements. A close association between DNA methylation and transcription level was identified for genes that participate in callus formation and shoot regeneration. Finally, we found that application of the DNA methyltransferase inhibitor 5′-Aza reduced both callus formation and shoot regeneration from leaf explants, which may have resulted from decreased transcript levels of genes related to cell wall integrity.

### Dynamic DNA methylation during callus formation

Previous studies have demonstrated the changes in chromatin structure associated with *in vitro* cell fate switching, represented by modulations of DNA methylation, histone modification, and small RNAs [19]. Using leaves and leaf-derived callus as materials, we found an overall hyper-methylation during callus formation. Consistent with our results, the induction of hypermethylation during callus formation has been reported in maize, *Populus trichocarpa*, and sugar beet using immature embryos, internodes, and leaves as explants, respectively [18, 28, 29]. On the other hand, we observed an increase in mCG and mCHG and a decrease in mCHH during callus formation in woodland strawberry, different from results in some other plant species. For example, mCG methylation does not change, mCHG increases, and mCHH decreases during callus formation from leaf explants in Arabidopsis [17].

Our data reveal that the mCG level decreases slightly in TE bodies and increases in TE-adjacent sequences during callus formation, whereas mCHG and mCHH decrease significantly in both TE bodies and surrounding sequences. Although it is generally believed that TEs are hypomethylated and activated during the process of callus formation [30, 31], DNA methylation patterns vary among different plants species. Zakrzewski et al. described an overall increase in DNA methylation of TEs in all CG, CHG, and CHH sequence contexts in leaf-derived callus of sugar beet [18], different from the pattern we observed in strawberry. Shim et al. reported that mCG levels showed negligible change in TE regions, mCHG levels increased, and mCHH levels decreased in callus tissues compared to leaves of Arabidopsis [17]. Thus, hypomethylation of TEs is not necessarily a feature of pluripotent callus cells derived from differentiated tissues. DNA methylation changes may be organ- and species-specific and may vary depending on the degree of differentiation of the explants.

### Association between DNA methylation and transcript level of genes essential for callus formation

DNA methylation within the gene promoter can affect transcription by altering chromatin structure and blocking or promoting the binding of transcription factors (both activators and repressors) [32]. On the other hand, DNA methylation in the gene body is usually correlated with gene transcription activity [33, 34]. The impact of DNA methylation on gene transcription is complicated in plants, and there is a lack of evidence for a general positive or negative correlation between DNA methylation and gene transcript levels. In this study, we observed that overall DNA methylation levels differed among genes from silent, lower, medium, and higher expression groups in *F. vesca*. For genes expressed at medium and higher levels, although their transcript levels were negatively correlated with the DNA methylation level in the CG and CHG context in promoters, this correlation was weak.

As discussed above, our data indicate that the general causal effect of DNA methylation on gene expression during callus formation is not clear. However, when considering genes involved in callus formation and shoot regeneration, those that were differentially expressed during callus formation, including *FvePLT3/7*, *FvePLT5*, *FveWIND3*, *FveWIND4*, *FveWOX14*, and *FveRAP2.6 L*, were frequently associated with differentially methylated regions, primarily in their promoters. Previous studies have demonstrated a regulatory role for DNA methylation and histone modifications in organogenesis in Arabidopsis. Examples include the modification of *WUS*-associated shoot regeneration by the DNA methyltransferase MET1 [21, 22] and the regulation of *WIND1* and *ERF113/RAP2.6 L* by histone acetylation during wound-induced cellular reprogramming [35]. Thus, epigenetic factors, including DNA methylation and histone modifications, may be general factors that modulate callus formation and regeneration capacity in plants.

### Callus formation is blocked by a DNA methyltransferase inhibitor

The drug 5′-Aza is an analog of cytidine that blocks the enzymatic activity of DNA methyltransferase [36]. We observed that blocking DNA methyltransferase activity by 5′-Aza inhibited callus and shoot formation from leaf explants of *F. vesca*. Moreover, 17 out of the 204 genes downregulated by 5′-Aza are involved in cell wall metabolism, including *expansins*, *xyloglucan endotransglucosylases*, *pectin lyases*, and *pectin methylesterases*, indicating the involvement of cell wall modification and integrity in callus formation. Callus initiation is a process similar to lateral root emergence [37], which is associated with changes in cell wall composition and structure [38, 39]. Thus, the negative effect of 5′-Aza on callus formation and shoot regeneration may be caused by the reduced transcription of genes related to cell wall integrity.

The effect of DNA methyltransferase inhibitors on the organogenesis pathway varies among different species. Previous studies have reported that 5′-Aza inhibits callus formation in maize and tobacco [40] but induces cell death in *Brachypodium distachyon* and sugarcane [41, 42]. In addition, several lines of evidence demonstrate that 5′-Aza promotes shoot regeneration in some species [43] but inhibits regeneration in others [44]. The process of callus formation and shoot regeneration is coupled with remarkable changes in gene transcription in a stage- and cell line-specific manner. The effect of drug treatment may depend on the time point and duration of drug application and on cell line-specific changes in the DNA methylome and transcriptome upon disturbance of DNA methylation.

Callus formation and shoot regeneration are essential for plant biotechnology. DNA methylation and other epigenetic factors have been emerging as important regulators of the acquisition of pluripotency and regeneration. This study reveals the dynamic DNA methylomes during callus formation from strawberry leaves and demonstrates the involvement of DNA methylation in this process. Our data provide clues for the improvement of regeneration systems for molecular breeding of strawberry and other Rosaceae species.

## Materials and methods

### Plant materials

Seeds of the woodland strawberry *Fragaria vesca* (Rugen) were surface-sterilized and planted in MS medium, then cultivated in a growth chamber under a 16-h light and 8-h dark photoperiod at 22°C. Leaves (30–35 d old) from 50–60-d-old seedlings were cut and cultivated on callus induction medium (MS-B5 with 0.2 μM IBA and 2 μM TDZ). The leaf explants were cultured in the dark for 3 d, then transferred to low-light conditions (250 lux, 22°C). After four weeks, the callus growing along the cut edges was carefully separated from the leaf explants and flash-frozen in liquid nitrogen.

### Treatment with 5′-azacytidine

The DNA methyltransferase inhibitor 5-azacytidine (Sigma-Aldrich, a2385-1 g) was applied to the induction medium at concentrations of 5, 10, and 20 μM. Leaf explants cultured on the induction medium were placed in a growth chamber (22°C, low light conditions). The leaf explants were transferred to fresh medium every 15 d, and the callus growth process was recorded.

To collect 5′-Aza-treated leaf materials for RNA-seq and BS-seq, the explants were cultured in liquid MS-B5 medium with 0 (control) or 20 μM 5′-Aza (treatment) for 20 min on a shaker at 100 rpm. Then the leaf explants were spread onto induction medium with or without 20 μM 5′-Aza and placed in a growth chamber (22°C, low light conditions). The leaf explants were collected after 0.5 and 3 d.

### Whole-genome BS-seq and data analysis

DNA from the leaf and callus tissues was extracted by a modified CTAB method. Library preparation and high-throughput sequencing were performed by BGI (Shenzhen, China). In brief, 3 μg genomic DNA was sonicated to 100–300 bp and then adenylated. DNA fragments 300–400 bp in length were selected and treated with bisulfite using the Methylation-Gold Kit (ZYMO). PCR was performed, and 350–400 bp long DNA sequences were purified. The library was sequenced on the Illumina HiSeq X Ten platform (BGI, Wuhan, China), and each replicate finally produced more than 8 Gb of clean data. Each sample had two biological replicates.

The bisulfite sequencing data were mapped to the reference genome (*F. vesca* v4.0) using Bismark software (version 0.16.3) [45], and the results of methylation extractor were used for subsequent analysis.

DMRs were identified and analyzed using the callDMR function (minCG = 3, minlen = 50, p.threshold = 0.05, pct.sig = 0.5, dis.merge = 100 and mean methylation difference > 0.15) of DSS software [46]. DMR-associated genes (DMGs) were defined as those with DMRs located in their promoters (2 kb upstream of the TSS) or gene bodies. Hyper-methylation indicates that the average methylation frequency of a cytosine in callus is higher than that of the same cytosine in leaf tissue, and vice versa.

### RNA sequencing and data analysis

RNA was extracted using the CTAB-PBIOZOL reagent (Beijing Baiyi Xinchuang Technology Co, Ltd, Cat: BSC55M1) according to the manual instructions. RNA sequencing was performed on the BGISEQ-500 platform by BGI.

The sequencing data were filtered using SOAPnuke (v1.5.2) [47], and the clean reads were aligned to the reference genome using HISAT2 v2.1.0 with default parameters (https://www.rosaceae.org/) [48]. FeatureCounts was used to calculate the number of reads that mapped to each gene. The transcript levels of each gene were normalized by the expected number of Fragments Per Kilobase of transcript sequence per Million base pairs sequenced (FPKM) method. The edgeR package (1.18.0) was used to perform the differential expression analysis [49], and *P* values were adjusted based on the method of Benjamini and Hochberg to control the false discovery rate (FDR). Genes with *P_adj_* < 0.05 and fold change ≥2 were regarded as DEGs. The GO and KEGG enrichment analyses were performed using OmicShare tools (www.omicshare.com/tools) for data analysis.

## Acknowledgements

This work was supported by the National Natural Science Foundation of China (31672123 to TG).

## Data Availability

The original data used to support the findings in this paper can be obtained by contacting the corresponding author.

## Conflict of interests

We declare no competing interests.

## Supplementary Material

Web_Material_uhab073
