# Supplementary material for: The callus formation capacity of strawberry leaf explants is modulated by DNA methylation
Source: Hortic Res. 2022 Jan 19;9:uhab073. doi: 10.1093/hr/uhab073 (PMC8947209; doi:10.1093/hr/uhab073)
Supplement: Web_Material_uhab073 [file web_material_uhab073.zip › Supplementary data Figures.pptx]

## Slide 1
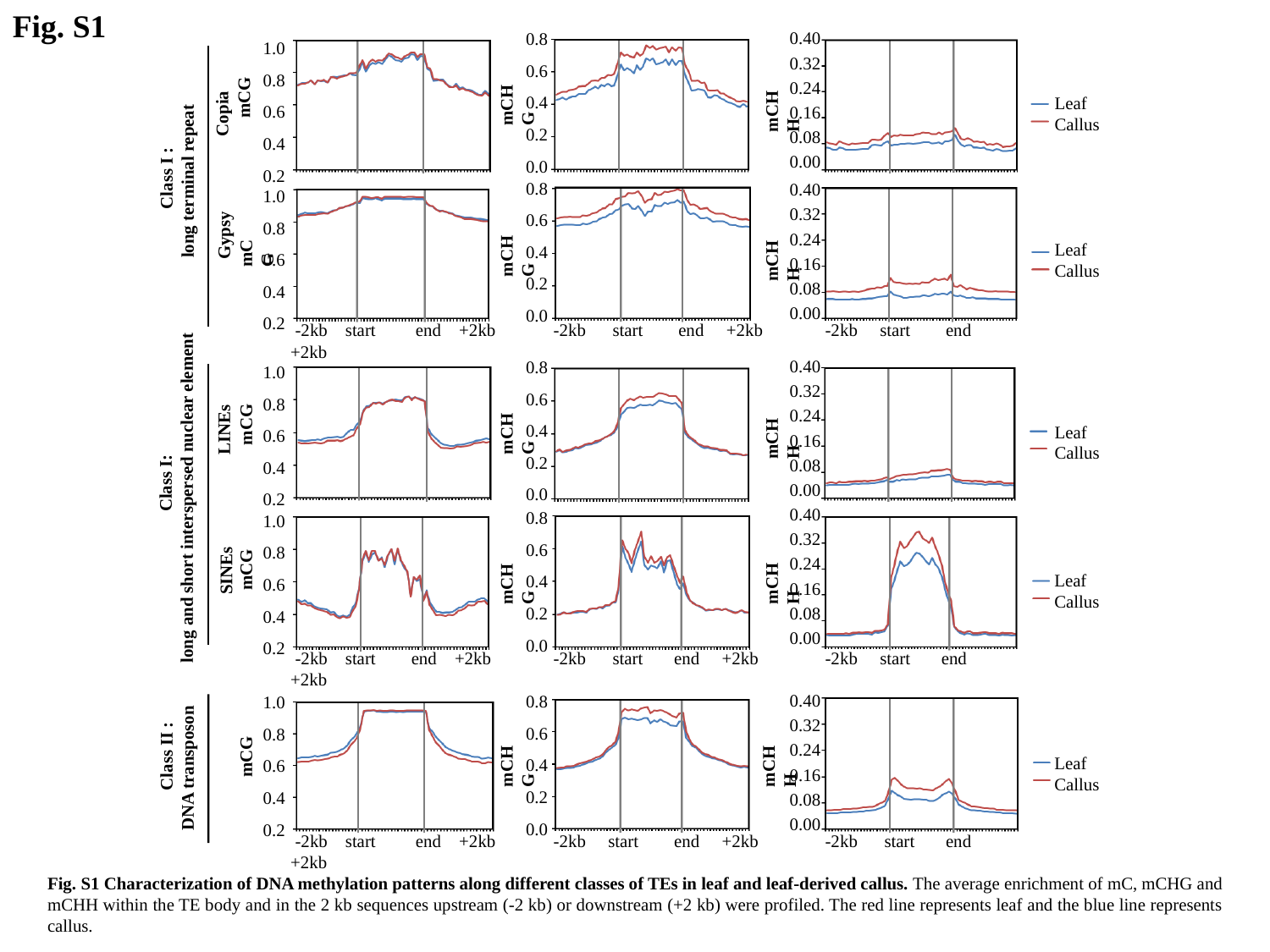

Fig. S1
0.8
0.6
0.4
0.2
0.0
0.40
0.32
0.24
0.16
0.08
0.00
1.0
0.8
0.6
0.4
0.2
mCG
mCHG
Copia
 Class I :
long terminal repeat
0.40
0.32
0.24
0.16
0.08
0.00
1.0
0.8
0.6
0.4
0.2
Gypsy
mCG
mCHG
 -2kb start end +2kb -2kb start end +2kb -2kb start end +2kb
1.0
0.8
0.6
0.4
0.2
mCG
mCHG
LINEs
 Class I:
 long and short interspersed nuclear element
1.0
0.8
0.6
0.4
0.2
mCG
mCHG
SINEs
 -2kb start end +2kb -2kb start end +2kb -2kb start end +2kb
1.0
0.8
0.6
0.4
0.2
mCG
mCHG
 Class II :
DNA transposon
 -2kb start end +2kb -2kb start end +2kb -2kb start end +2kb
Leaf
Callus
Leaf
Callus
Leaf
Callus
Leaf
Callus
Leaf
Callus
mCHH
mCHH
mCHH
mCHH
mCHH
0.8
0.6
0.4
0.2
0.0
0.8
0.6
0.4
0.2
0.0
0.40
0.32
0.24
0.16
0.08
0.00
0.8
0.6
0.4
0.2
0.0
0.40
0.32
0.24
0.16
0.08
0.00
0.8
0.6
0.4
0.2
0.0
0.40
0.32
0.24
0.16
0.08
0.00
Fig. S1 Characterization of DNA methylation patterns along different classes of TEs in leaf and leaf-derived callus. The average enrichment of mC, mCHG and mCHH within the TE body and in the 2 kb sequences upstream (-2 kb) or downstream (+2 kb) were profiled. The red line represents leaf and the blue line represents callus.

## Slide 2
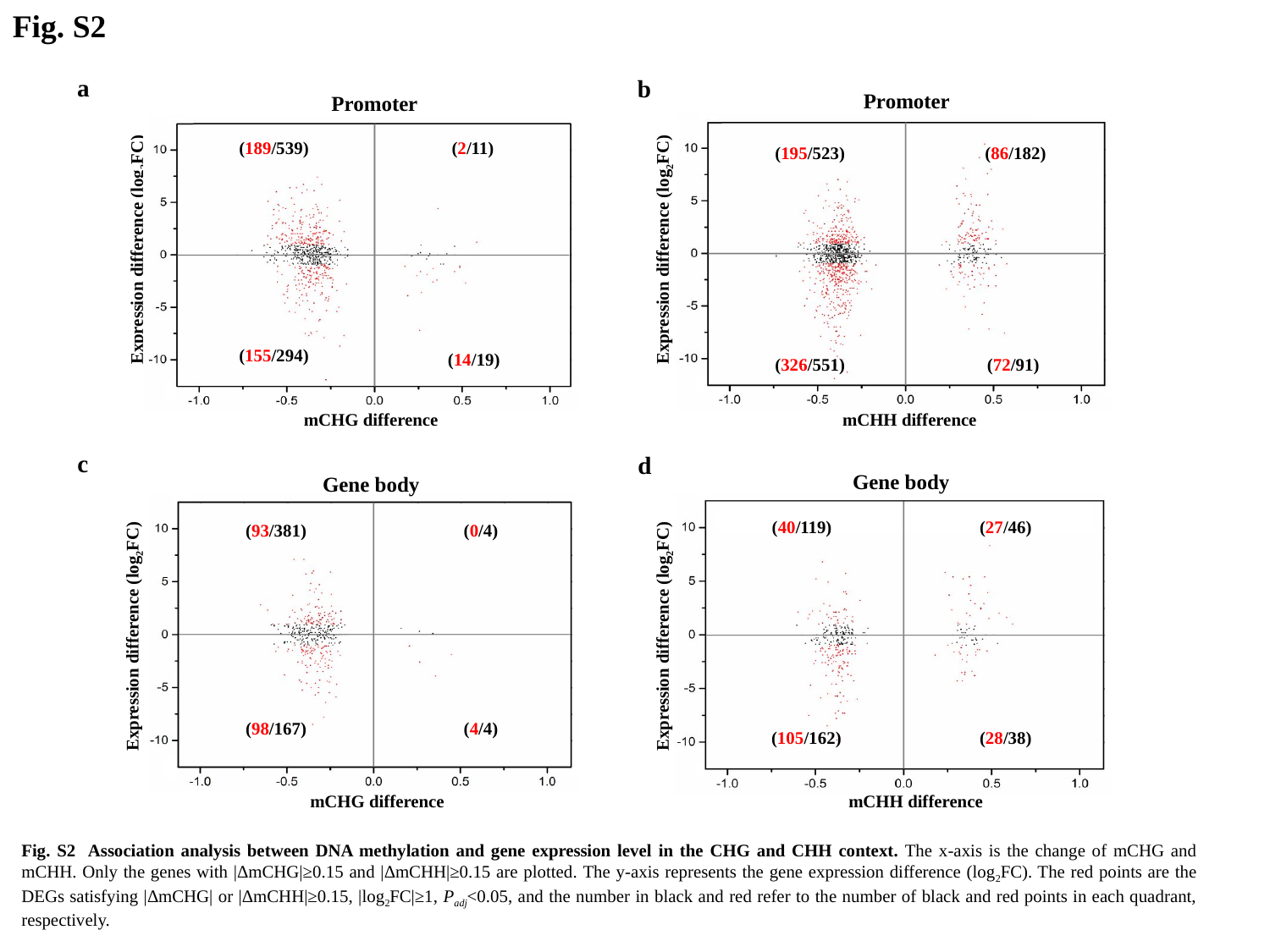

Fig. S2
a
b
 Promoter
 Promoter
 (189/539)
 (2/11)
 (195/523)
 (86/182)
Expression difference (log2FC)
Expression difference (log2FC)
 (155/294)
 (14/19)
 (326/551)
 (72/91)
mCHG difference mCHH difference
c
d
 Gene body
 Gene body
 (40/119)
 (27/46)
 (93/381)
 (0/4)
Expression difference (log2FC)
Expression difference (log2FC)
 (98/167)
 (4/4)
 (105/162)
 (28/38)
mCHG difference mCHH difference
Fig. S2 Association analysis between DNA methylation and gene expression level in the CHG and CHH context. The x-axis is the change of mCHG and mCHH. Only the genes with |ΔmCHG|≥0.15 and |ΔmCHH|≥0.15 are plotted. The y-axis represents the gene expression difference (log2FC). The red points are the DEGs satisfying |ΔmCHG| or |ΔmCHH|≥0.15, |log2FC|≥1, Padj<0.05, and the number in black and red refer to the number of black and red points in each quadrant, respectively.

## Slide 3
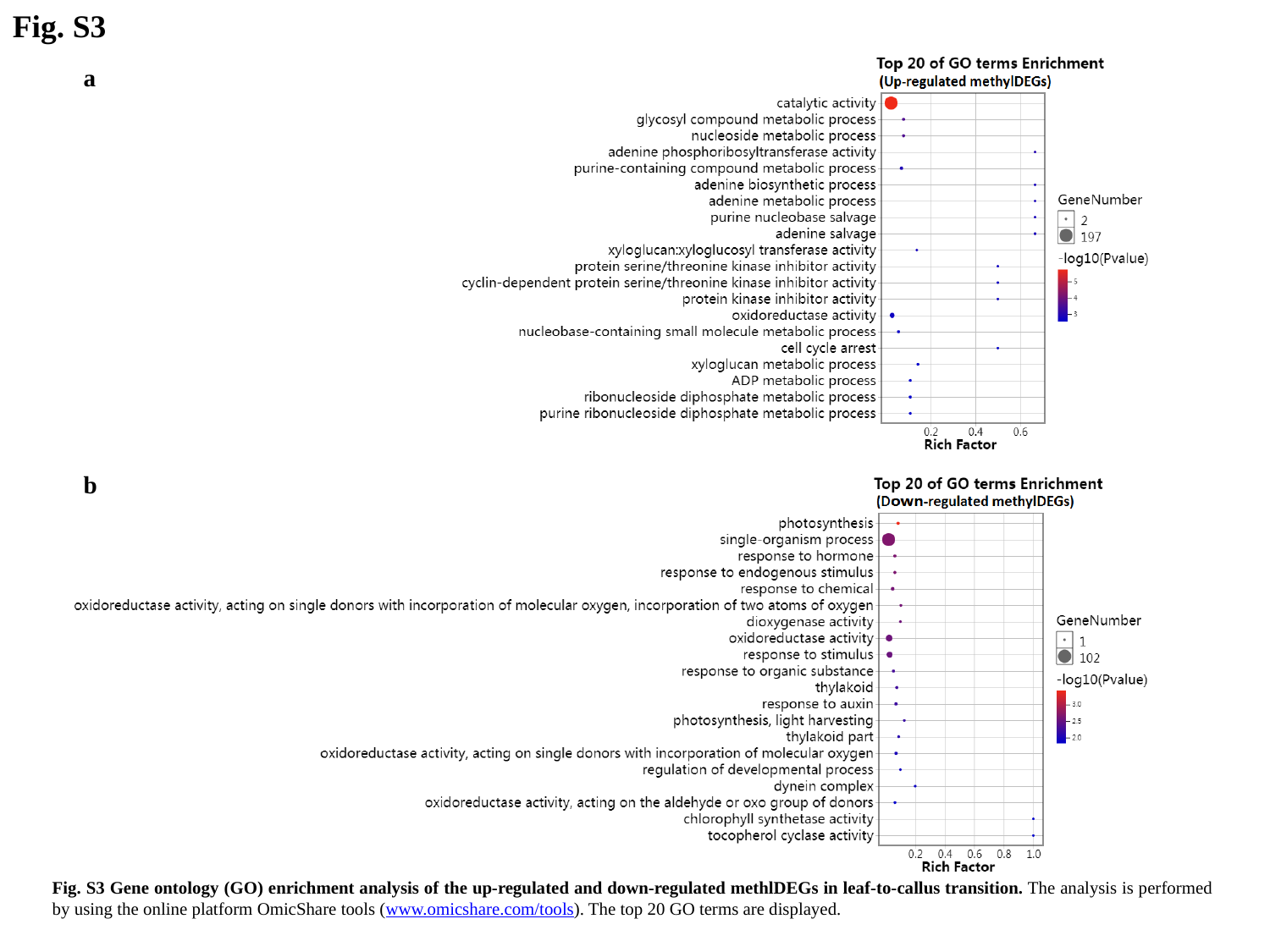

Fig. S3
a
b
Fig. S3 Gene ontology (GO) enrichment analysis of the up-regulated and down-regulated methlDEGs in leaf-to-callus transition. The analysis is performed by using the online platform OmicShare tools (www.omicshare.com/tools). The top 20 GO terms are displayed.

## Slide 4
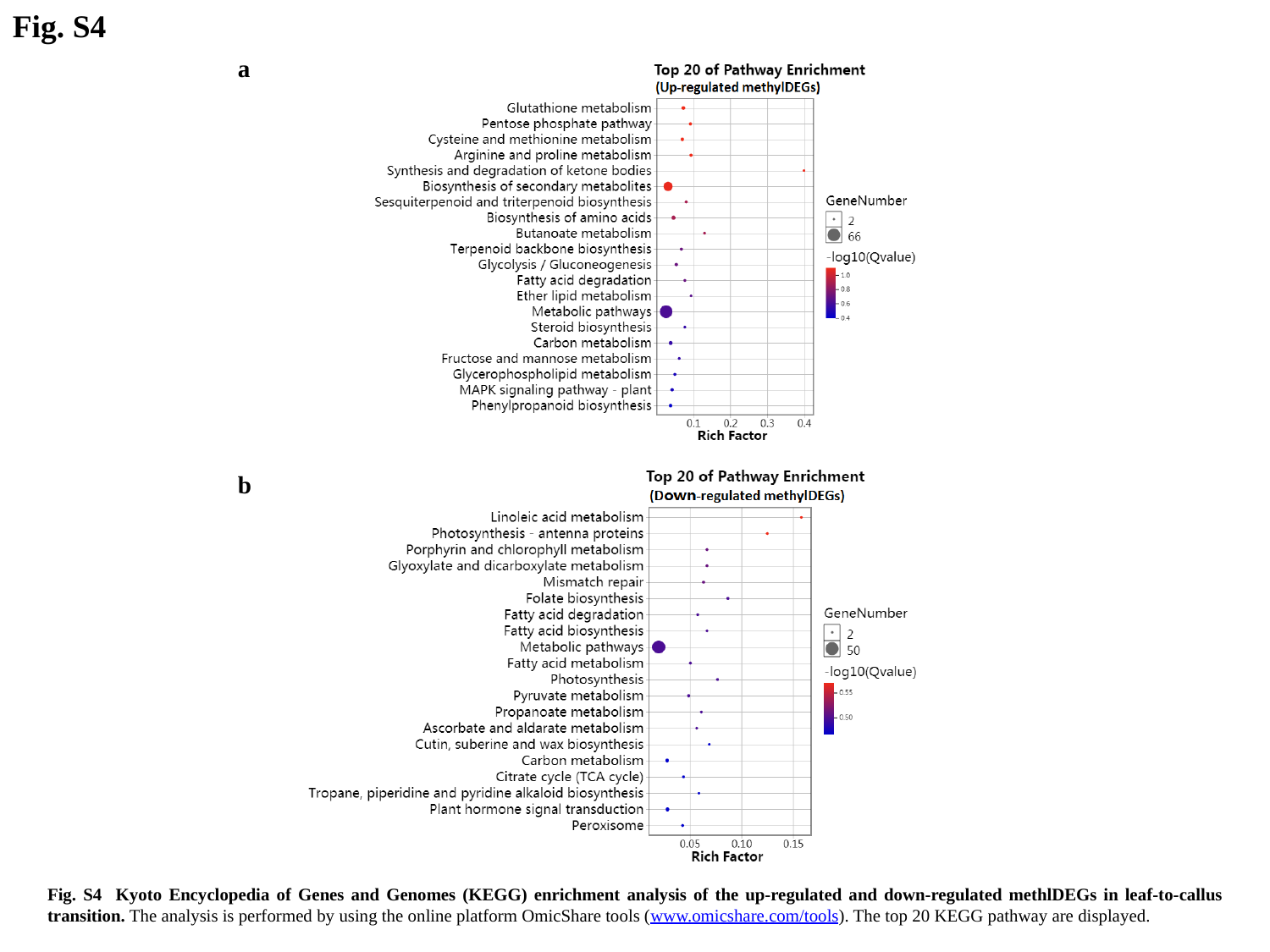

Fig. S4
a
b
Fig. S4 Kyoto Encyclopedia of Genes and Genomes (KEGG) enrichment analysis of the up-regulated and down-regulated methlDEGs in leaf-to-callus transition. The analysis is performed by using the online platform OmicShare tools (www.omicshare.com/tools). The top 20 KEGG pathway are displayed.

## Slide 5
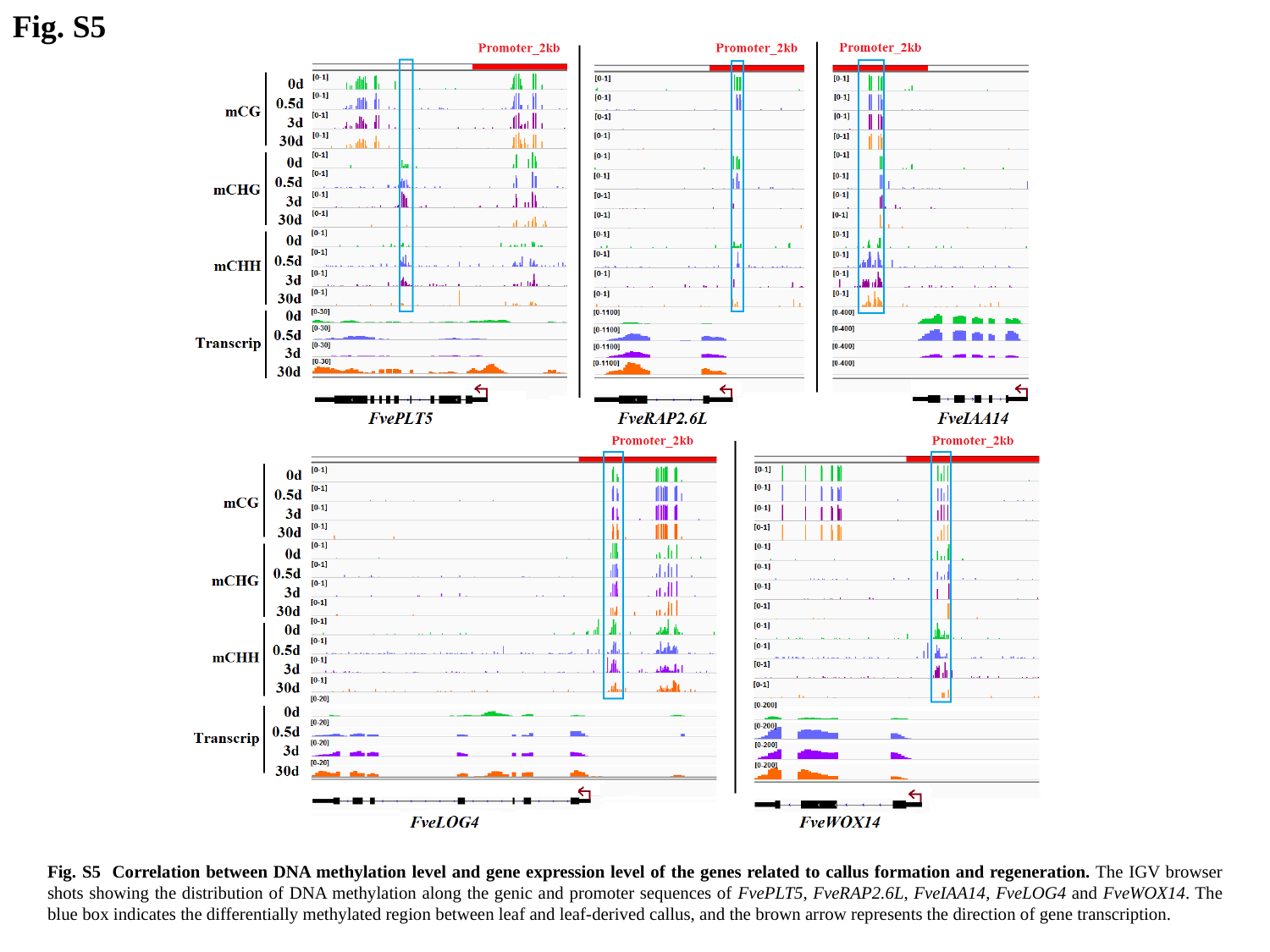

Fig. S5
Fig. S5 Correlation between DNA methylation level and gene expression level of the genes related to callus formation and regeneration. The IGV browser shots showing the distribution of DNA methylation along the genic and promoter sequences of FvePLT5, FveRAP2.6L, FveIAA14, FveLOG4 and FveWOX14. The blue box indicates the differentially methylated region between leaf and leaf-derived callus, and the brown arrow represents the direction of gene transcription.
